# Music Listening as a Strategy for Managing COVID-19 Stress in First-Year University Students

**DOI:** 10.3389/fpsyg.2021.647065

**Published:** 2021-04-01

**Authors:** Dianna Vidas, Joel L. Larwood, Nicole L. Nelson, Genevieve A. Dingle

**Affiliations:** ^1^UQ Music, Dance & Health Research Group, The University of Queensland, St Lucia, QLD, Australia; ^2^School of Psychology, The University of Adelaide, Adelaide, SA, Australia

**Keywords:** music listening, stress, coping, University students, international students, emotion

## Abstract

The COVID-19 pandemic brought rapid changes to travel, learning environments, work conditions, and social support, which caused stress for many University students. Research with young people has revealed music listening to be among their most effective strategies for coping with stress. As such, this survey of 402 first-year Australian University students (73.9% female, *M*_age_ = 19.6; 75% domestic and 25% international) examined the effectiveness of music listening during COVID-19 compared with other stress management strategies, whether music listening for stress management was related to well-being, and whether differences emerged between domestic and international students. We also asked participants to nominate a song that helped them to cope with COVID-19 stress and analyzed its features. Music listening was among the most effective stress coping strategies, and was as effective as exercise, sleep, and changing location. Effectiveness of music listening as a coping strategy was related to better well-being but not to level of COVID-19 related stress. Although international students experienced higher levels of COVID-19 stress than domestic students, well-being was comparable in the two cohorts. Nominated songs tended to be negative in valence and moderate in energy. No correlations were found between any self-report measure and the valence and energy of nominated coping songs. These findings suggest that although domestic and international students experienced different levels of stress resulting from COVID-19, music listening remained an effective strategy for both cohorts, regardless of the type of music they used for coping.

## Introduction

During the 2020 COVID-19 pandemic, Australian Government health legislation forced individuals into different ways of working, studying, and living to control the spread of the virus. University students were one population severely impacted by these measures. Not only were traditional lectures and classes affected as learning moved online, but opportunities for students to socialize, travel, and work were also interrupted. International students may have been particularly affected; many had to return home, or stay in their host country, physically distanced from friends and family for an uncertain period of time. It is estimated that more than 100,000 students who planned to study in Australia in 2020 were unable to travel from China (Ziguras and and Tran, 2020). The pandemic brought about novel stressors and potentially amplified existing stressors, requiring students to select and employ a variety of coping strategies. Previous research has shown music to be a common stress coping strategy among young people (Groarke and Hogan, 2019; Dingle et al., 2020), but whether strategies such as music listening are effective for domestic and international students in Australia under the circumstances of a pandemic remain to be seen.

Initial studies conducted during the pandemic are positive on this score, and not just due to viral COVID-19 songs (Lehman, 2020). While in lockdown, Spanish people spent more time devoted to musical activities to cope, and found that music listening helped them relax, escape, raise their mood, and keep them company (Cabedo-Mas et al., 2021). In Australia, life-satisfaction was positively associated with music listening, and negatively associated with other activities, such as TV watching (Krause et al., 2021), highlighting that music use may be a crucial tool during the lockdowns caused by COVID-19. Several other papers have highlighted that music has been an effective tool to cope with psychological distress during the pandemic, as a proxy for social interaction, and to improve well-being during the pandemic (Mas-Herrero et al., 2020; Fink et al., 2021; Granot et al., 2021). Interestingly, lockdowns and COVID-19 incidence also appeared to impact on the music listened to by Spotify users, such that nostalgic songs appeared prominently in daily charts during phases of lockdown in several European countries (Yeung, 2020).

On average, adults listen to music for at least an hour a day, and, in the case of younger adults, up to 18 h a week (Krause et al., 2015; Papinczak et al., 2015). In an Australian sample, music listening has been rated via self-report as one of the most effective stress management strategies, along with watching television/movies, spending time with friends and family, focusing on positives, and reading, among others (Casey and Liang, 2014). Thayer et al. (1994) similarly found that exercise, listening to music, social interaction, tending to chores, and sleep were the most successful techniques to change a bad mood, while to reduce tension, the most successful strategies included religious activity, listening to music, tending to chores, exercise, and social interaction. Evidently, music listening, among other strategies can be effective for a variety of mood regulation situations.

Among the most common reasons for listening to music are generating and regulating emotion experiences (Västfjäll et al., 2012; McFerran et al., 2015; Papinczak et al., 2015), and music is used for emotional self-regulation throughout adulthood (Saarikallio, 2011). Importantly, this is evident across multiple cultures (Boer and Fischer, 2011; Boer et al., 2012; Schäfer et al., 2012; Chin, 2016). Emotion regulation is one of several functions of music listening. It is a continuous process, with a range of contextual and individual factors that play a role in how music may link with well-being (Baltazar, 2019; Baltazar and Saarikallio, 2019). Furthermore, through its effects on emotions, music listening has been found to benefit well-being among listeners of all ages (Papinczak et al., 2015; Groarke and Hogan, 2016).

When coping with stress, previous research has often concentrated on the concept that soft, slow music, such as classical music, is better for managing negative emotions compared with hard or heavy music (Burns et al., 1999, 2002; Labbé et al., 2007). However, the evidence for this claim is mixed. For instance, the type of music listened to may not strongly influence relaxation (Davis and Thaut, 1993). Research has shown that people experiencing pain tend to select music that is higher in energy and danceability, and less instrumental, than the music chosen by experimenters (Howlin and Rooney, 2020). Individuals often listen to sad-sounding music when sad, and after experiencing negative events, although the evidence is mixed on whether this is beneficial for listeners (Garrido and Schubert, 2013; van den Tol, 2016; Larwood and Dingle, 2021). Individuals also choose to listen to angry music when upset (McFerran et al., 2015), and fans of extreme metal experience positive emotions when listening to death metal music (Thompson et al., 2018) and other extreme music (Sharman and Dingle, 2015). Importantly, self-selected music may be more effective for regulating negative emotions compared with experimenter-selected music (Groarke and Hogan, 2019). Furthermore, in the context of COVID-19, a receptive music therapy study showed that playlists customized to clinical staff working with COVID-19 patients had a greater impact than pre-made “relaxation,” “energy,” and “serenity” playlists (Giordano et al., 2020). Thus, people experiencing negative emotional states may choose to listen to a variety of music, particularly mood congruent music, and this may be beneficial in managing their emotions and improving their well-being (Papinczak et al., 2015; Randall and Rickard, 2017).

## Aims, Research Questions, and Hypotheses

This study was designed to examine the effectiveness of music listening for managing stress and supporting well-being during COVID-19. Australian domestic and international first-year students were surveyed about their stress, coping strategies, and well-being during Australia's first wave of COVID-19, in the period from March to July 2020, and asked to nominate a specific song that helped them cope with stress. These songs were analyzed with the SpotifyR package, which allowed for investigation of musical features such as valence (positivity), energy, tempo, danceability, and other features of songs (Thompson et al., 2019). The study had two specific hypotheses and two exploratory questions:

H1. In line with previous research in young people, and due to the accessibility of music listening during COVID-19; we hypothesized that music listening would be rated as the most effective stress management strategy among this University student population in the context of COVID-19.

H2. We also hypothesized that music listening effectiveness would be related to participants' well-being during COVID-19.

Exploratory research questions examined whether domestic and international students experienced differences in levels of stress and in the use of music listening for managing stress. Finally, the study explored features of the music that students nominated as helping them to manage stress during COVID-19.

## Methods

### Participants

Participants were 402 first year students enrolled in a variety of degree programs in a major metropolitan University in Queensland, Australia. Data were adequately sampled (>50% items completed) from 303 domestic students (*M*_age_ = 19.1, *SD*_*age*_ = 4.10, 73.9% female, 25.40% male) and from 99 international students (*M*_age_ = 20.1, *SD*_*age*_ = 5.46, 73.7% female, 25.25% male). Most domestic students were Australian citizens (76.2%), while 15.5% were dual citizens of Australia and another country. For the international students, 19.2% were Chinese citizens, 15.2% were citizens of Hong Kong, 13.1% were citizens of Singapore, and 10.1% were citizens of Indonesia. The remaining 57% of students were from several other countries of origin, predominantly in Asia.

### Measures

#### COVID-19 Related Stress

Participants indicated their levels of stress related to COVID-19 on a purpose-built measure of 13 items developed by members of the broader project. Questions were taken to a small group of student advisors, predominantly international students, to collaboratively determine face validity for assessing stress due to COVID-19 for domestic and international students. The COVID-19 stress scale showed good internal consistency, α = 0.857. Participants rated the extent to which a variety of issues were causing them stress (e.g., travel restrictions; worry about running out of medical supplies and other supplies/groceries; worry about family members being infected). Items were rated on a 1 (strongly disagree) to 5 (strongly agree) scale, with higher summed scores indicating greater levels of COVID-19 related stress. Total scores ranged from 13 to 65.

#### Coping Strategies

Participants rated how often they found a list of 15 stress management strategies (i.e., listening to music; exercise; calling someone on the phone) to be effective. This list of activities was adapted from Thayer et al. (1994) with participants rating each on a 1 (none of the time) to 5 (all of the time) scale. Participants who did not engage in the activity were instructed to rate the item as 0 and their score was not used in the calculation of item means.

#### Well-Being

Participant well-being was measured using the Short Warwick Edinburgh Mental Well-being Scale (α = 0.86) (SWEMWBS; Stewart-Brown et al., 2009). The scale comprised of seven positively worded items (i.e., feeling useful; feeling close to other people), which participants rated on a 1 (never) to 5 (all of the time) scale, for the past 2 weeks. Scores were summed to produce a measure of well-being, with higher scores representing higher subjective well-being. Published norms from a UK sample produced a mean of 23.57, and standard deviation of 3.61 from a sample of 1143 16–24 year olds (Ng Fat et al., 2017).

#### Coping Music

Participants were asked to nominate a song they listened to that helped to manage their stress. Measures of the song's valence (positivity), energy, tempo, mode, danceability, acousticness, and instrumentalness were obtained by querying the API provided by Spotify AB. These variables, with the exception of mode and tempo, are scored from 0 to 1. Mode is dichotomous (1 indicates major, 0 indicates minor), and tempo is the estimated beats per minute. Data were retrieved using the Spotify API for 271 of the 402 participants (206 domestic, 65 international).

### Procedure

Upon providing consent, participants completed initial demographic questions (age, gender, domestic/international student status) before completing a battery of questionnaires. The questionnaire battery included the above measures in addition to measures relating to social connections and racial discrimination that were not analyzed as part of the current study. Participants took part in the study from late April to early June 2020. At this time, Queensland state borders were closed, stay-at-home rules were enforced, businesses such as gyms, cinemas and restaurants were closed, primary and secondary schools were closed to students until mid-May, and only virtual University attendance was permitted. Many international students returned home for the remainder of semester, and those in Australia had no access to the monetary support offered by the government to domestic students. In early May, restrictions began to ease, such that small gatherings were permitted, and recreational travel in-state was allowed, although state borders remained closed. The measures and procedure were approved by the University of Queensland Health and Behavioral Sciences Low and Negligible Risk Ethics Committee.

## Results

### Effectiveness of Music as a Coping Strategy and Well-Being

**Figure 1:**
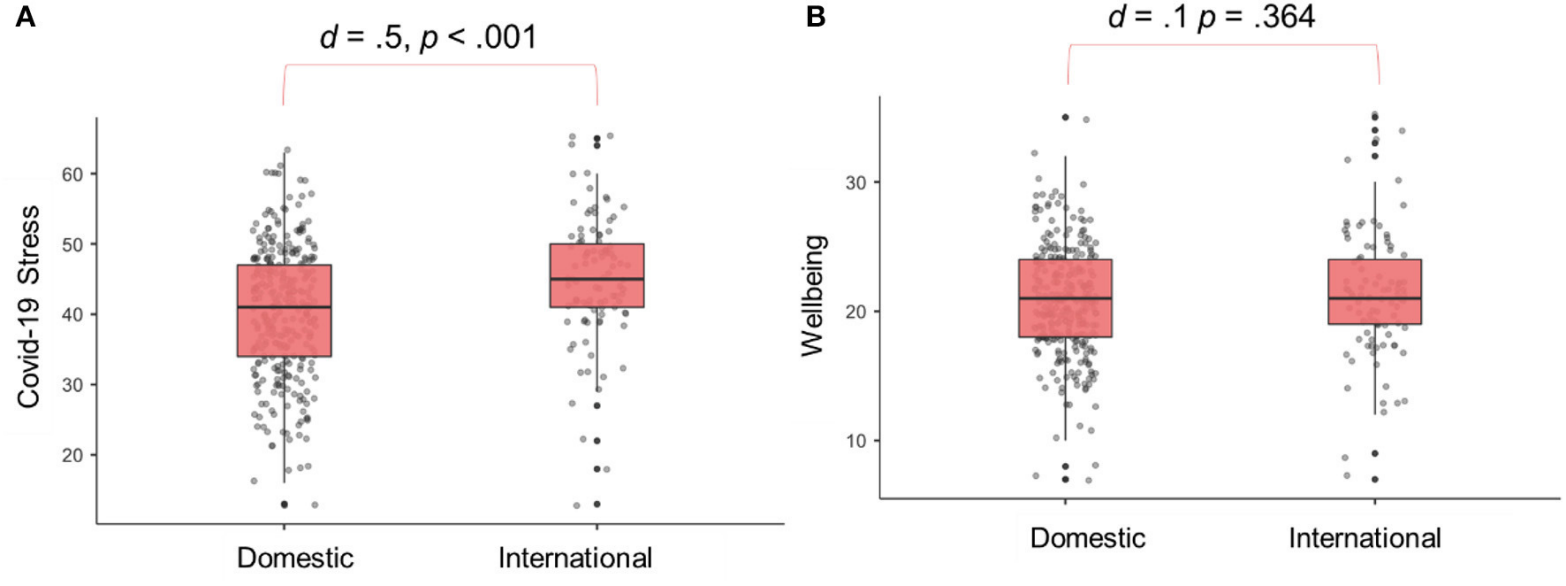
Box plots showing the distribution of COVID-19 Stress **(A)** and Short Warwick Edinburgh Mental Well-being Scale **(B)** for Domestic and International Students.

International students experienced greater COVID-19 related stress than domestic students, *t*(399) = 4.28, *p* < 0.001, *d* = 0.50 (Figure 1). Despite this, no difference was found between domestic students (*M* = 20.7, *SD* = 4.19) and international students (*M* = 21.2, *SD* = 4.89) on well-being, *t* = 0.91, *p* > 0.394, *d* <0.11. Figure 1 shows these distributions. Comparing transformed means to the norms presented in (Ng Fat et al., 2017) for 16–24 year old residents of the UK, our sample had lower well-being, for both domestic and international student groups (*p*s < 0.001). Additionally, when compared with a similar cohort of students from 2019 (Dingle et al., 2020, preprint), well-being was lower in 2020 for both domestic, *t*(538) = 6.61, *p* < 0.001, and international students, *t*(247) = 2.78, *p* = 0.006.

**Figure 2:**
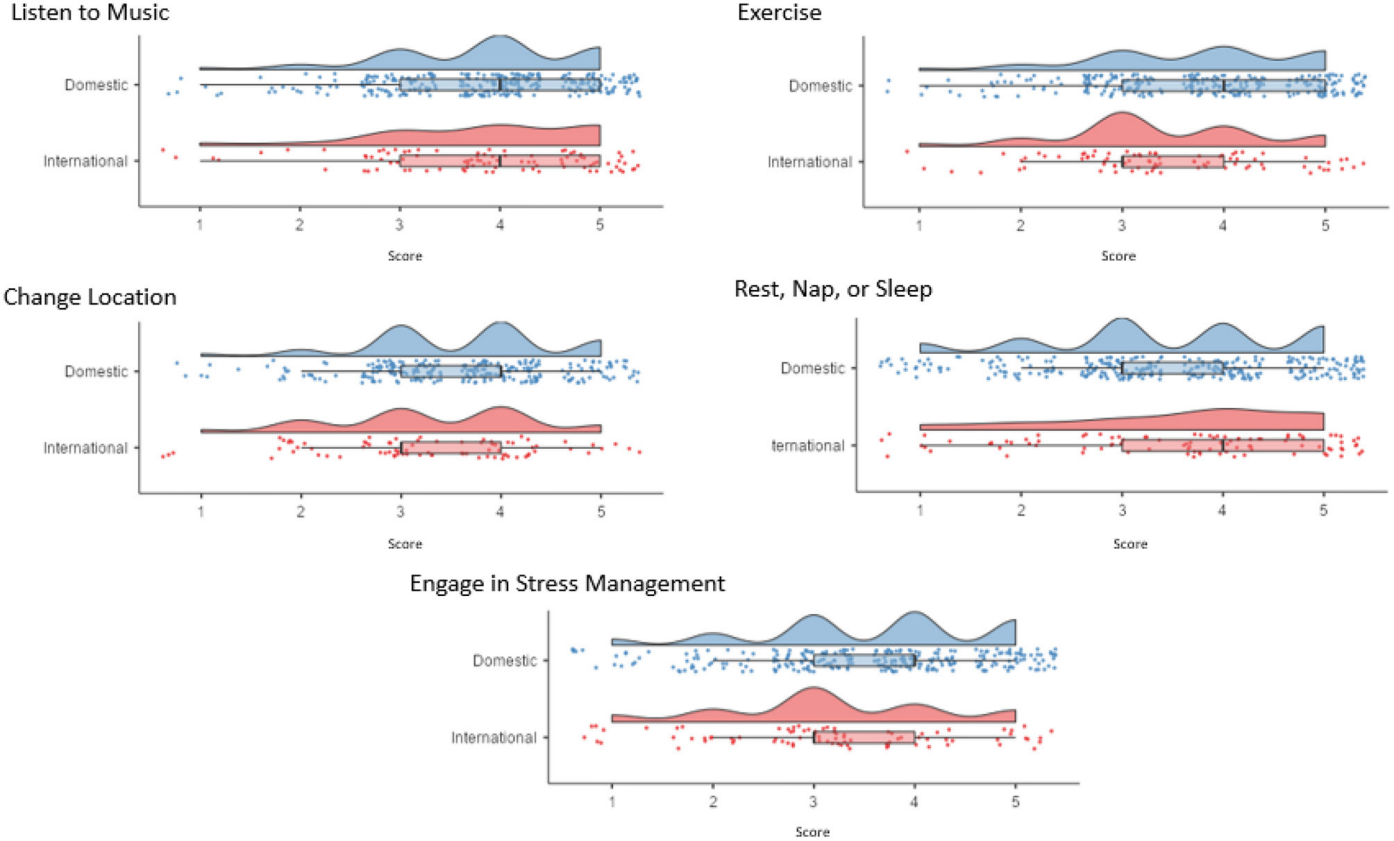
Box and violin plots showing the distribution of effectiveness ratings, for domestic and international students, for the top five methods of coping.

Music had the highest effectiveness rating of all coping strategies (*M* = 3.85, *SD* = 0.98), with no difference found in endorsement between domestic (*M* = 3.84, *SD* = 0.95) and international (*M* = 3.87, *SD* = 1.01) students, *t*(390) = 0.27, *p* = 0.79, *d* = 0.03. Holm corrections were used for all pairwise analyses. Exercise (*M* = 3.66, *SD* = 1.03), changing location (*M* = 3.54, *SD* = 0.96), and sleep (*M* = 3.48, *SD* = 1.21), were the second, third, and fourth most endorsed strategies, respectively, and did not significantly differ from music, *t*s <2.85, *p*s > 0.224 (Figure 2). This did not support Hypothesis 1, that music listening would be the most effective stress management strategy during COVID-19.

Pearson's correlations revealed that COVID-19 related stress was linked with reduced subjective well-being, *r* = −0.12, *p* = 0.017, but not endorsement of music as a coping strategy, *r* = 0.04, *p* = 0.477. Subjective well-being, however, was related to endorsement of music as an effective coping strategy, *r* = 0.13, *p* = 0.010. These data partly support the second hypothesis: how effective students found music as a coping strategy was slightly related to their well-being, although it was unrelated to their COVID-19 related stress.

### Coping Songs

Finally, we investigated the relationship between endorsement of music used as a coping strategy and computationally derived ratings of valence, energy, tempo, and mode (Spotify, 2019). Pearson's correlations showed no relationship between the endorsement of music as a coping strategy and musical features, *ps* > 0.101 or between student status and music features, *p*s > 0.589. Musical valence, energy, and tempo were unrelated to well-being, COVID-19 stress, and endorsement of music listening as an effective coping strategy (see Table 1).

**Table 1:** Pearson's correlations for musical features and well-being.

	**Effectiveness of music listening**	**COVID stress**	**Well-being**	**Energy**	**Valence**	**Tempo**	**Danceability**	**Acousticness**
COVID stress	0.036	–						
Well-being	0.133^**^	−0.121^*^	–					
Energy	0.008	0.014	−0.018	–				
Valence	0.050	< −0.001	0.076	0.487^***^	–			
Tempo	0.032	0.032	0.020	0.267^***^	0.074	–		
Danceability	0.022	0.026	0.079	0.334^***^	0.598^***^	0.101	–	
Acousticness	−0.012	0.089	0.015	−0.789^***^	−0.405^***^	−0.339^***^	−0.335^***^	–
Instrumentalness	−0.089	−0.025	−0.010	−0.503^***^	−0.367^***^	−0.190^**^	−0.377^***^	0.437^***^

* p < 0.05,

** p < 0.01,

*** *p < 0.001*.

**Figure 3:**
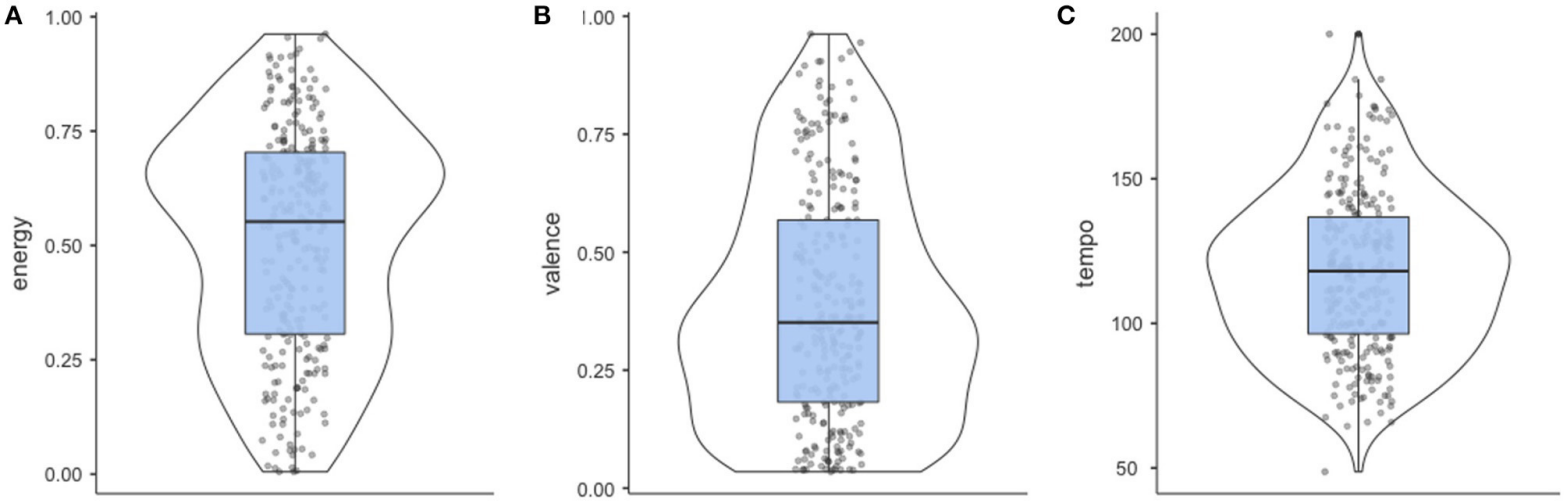
Distribution of continuous audio features for energy **(A)**, valence **(B)**, and tempo **(C)** in 271 songs nominated by University students as helping them cope with COVID-19 stress.

On a descriptive level, large amounts of variation were found in the retrieved musical features, with a significant skew found for the continuous measures of energy, valence, and tempo (see Figure 3). Musical energy was skewed (skewness = −0.27, *W* = 0.97, *p* < 0.001) with a mean value of 0.51 (*SD* = 0.25), such that energy was moderate. The valence of retrieved songs was also skewed (skewness = 0.44, *W* = 0.95, *p* < 0.001) with a mean rating of 0.39 (*SD* = 0.25), such that the average valence of reported songs was moderately negative. Tempo was also slightly skewed (skewness = 0.25, *W* = 0.99, *p* = 0.042) with a mean of 118 BPM (*SD* = 28.1 BPM). Retrieved songs were also likely to contain lyrics, with 87% of songs machine coded as likely containing lyrics*, p* < 0.001, and more likely to be in a major mode (71% of retrieved songs, *p* < 0.001) than a minor mode. These findings indicate that overall, the music listened to during COVID-19 tended to be moderately negative in valence, with a fast tempo, and energy above the midpoint. The songs listed by participants can be seen in the data file associated with this study (https://github.com/joellarwood/covid_music).

To contextualize these results, energy, valence, instrumentalness, danceability, valence, and tempo variables from the present study were compared with means for participant-selected music from Howlin and Rooney (2020), using a bonferroni corrected alpha (0.05/5) of 0.01. Table 2, compares these results. In our sample, self-selected music was lower in both energy, *t*(270) = −10.60, *p* < 0.001, *d* = 0.64 and valence *t*(270) = −8.17, *p* < 0.001, *d* = 0.50. In addition, music in our sample was more likely to be instrumental, *t*(270) = 2.84, *p* = 0.005, *d* = 0.17, and had comparable tempo, *t*(270) = 2.46, *p* = 0.014, and danceability, *t*(270) = 0.57, *p* = 0.564, to the music for pain in Howlin and Rooney's study (*p*s ≥ 0.014).

**Table 2:** Comparison of means and standard deviations for Howlin and Rooney (2020), and the present data, with associated *t*-tests.

	**Howlin and Rooney (2020)**	**Present data**	***t*-test results (corrected α = 0.01)**
Energy	0.67 (0.22)	0.51 (0.25)	*t*(270) = −10.6, *p* < 0.001, *d* = 0.64
Instrumentalness	0.08 (0.21)	0.13 (0.29)	*t*(270) = 2.84, *p* = 0.005, *d* = 0.17
Danceability	0.55 (0.17)	0.56 (0.18)	*t*(270) = 0.58, *p* = 0.56
Valence	0.51 (0.25)	0.39 (0.25)	*t*(270) = −8.17, *p* < 0.001, *d* = 0.50
Tempo	113.78 (31.27)	118 (28.1)	*t*(270) = 2.46, *p* = 0.014

## Discussion

This study aimed to explore the rated effectiveness and associated outcomes of music listening during the first wave of the COVID-19 pandemic in Australia. Our first hypothesis was that music listening would be rated as the most effective stress management strategy among University students in the context of COVID-19. This hypothesis was not supported. Music had the highest mean endorsement of all listed coping strategies, although it was not statistically different from exercise, sleep, and change of location. This was relatively consistent with top strategies reported in Thayer et al. (1994) for reducing tension, which included religious activity, listening to music, tending to chores, exercise, and social interaction. Importantly, music listening, among other strategies, remained an accessible and effective strategy for managing stress during times of stay-at-home orders and physical distancing. This finding was consistent with previous research demonstrating music listening to be an effective way of managing stress in a range of populations, methodologies, and situations (Thayer et al., 1994; Casey and Liang, 2014; Groarke and Hogan, 2019; Dingle et al., 2020). Furthermore, this finding corroborates with the growing COVID-19 literature, that has found that music is among top strategies for coping with psychological distress and to improve well-being (Mas-Herrero et al., 2020; Granot et al., 2021).

COVID-19 related stress and subjective well-being were related, such that higher COVID stress was related to slightly reduced well-being; however, differing patterns were observed in the mean difference between domestic and international students. In our sample, international students experienced higher levels of stress due to COVID-19 compared with domestic students. As the items used to measure COVID-19 stress largely related to practical and logistic considerations this difference is unsurprising and speaks to the unique difficulties faced by international students in the pandemic context. Well-being scores for domestic and international students in our sample were equitable, however the scores for both groups were significantly lower than existing (pre-pandemic) norms in young people from the UK (Ng Fat et al., 2017). This highlights the emotional consequences of the pandemic, regardless of student status, and associated difficulties presented for social life (Van Bavel et al., 2020).

Our second hypothesis was that music listening effectiveness would be related to participants' well-being during COVID-19. This was supported, with endorsement of music use for coping relating to slightly higher well-being. This re-affirms previous research on the benefits of music listening for well-being (Groarke et al., 2020; Howlin and Rooney, 2020), and is consistent with similar findings that music listening, unlike other leisure activities like watching TV, is positively associated with life satisfaction in the context of COVID-19 (Krause et al., 2021). That music listening remains effective even during the stress of a global pandemic emphasizes the potential of music during crisis or when ability to connect socially is limited.

Using the healthy and unhealthy uses of music as a guide (Saarikallio et al., 2015), it could be speculated that participants in this sample were not using music in a way that fosters rumination or avoidance, but were instead using music listening to foster feeling of connectedness and facilitate affective change during COVID-19. At the time of data collection, Australia was experiencing Government mandated social distancing policies and stay-at-home restrictions that limited physical social contact. Subsequently, music listening may have provided listeners with a way to feel connected with others by acting as a social surrogate and also providing feelings of solace or consolation (Hanser et al., 2016; Schäfer and Eerola, 2020; Schäfer et al., 2020).

An exploratory question for the study examined the kind of music University students listen to for managing stress related to COVID-19. Coping songs tended to be negative in valence, and moderate in energy—this is affectively similar to stress, a negatively valenced, high arousal emotion. The lack of correlation between the characteristics of the music listened to and rated endorsement of music as a coping strategy highlights the importance of self-selection of music, where self-selection respects the agency of the listener, and acknowledges that while slower, positively valenced music may be congruent with relaxation, it is not necessary for coping with negative emotions (Burns et al., 1999, 2002; Labbé et al., 2007; Groarke and Hogan, 2019; Groarke et al., 2020; Howlin and Rooney, 2020). Further supporting this notion, the expressed positivity, energy, and tempo of coping music was not related to well-being or COVID-19 stress.

To better contextualize our results, the songs listed by participants for coping with COVID-19 related stress were compared with music chosen by participants for coping with pain (Howlin and Rooney, 2020). This comparison allowed us to investigate trends across studies where individuals are in a broadly negative mood state. Songs in the present study were lower in both energy and valence than participant-choice music for coping with pain. This suggests that people who are choosing music to cope with stress choose quieter, less uplifting music, with a lower dynamic range and onset rate than people experiencing pain (Spotify, 2019; Howlin and Rooney, 2020). Furthermore, music chosen for coping with stress was more likely to be instrumental, but had comparable danceability and tempo than music selected to cope with pain. Although both groups were experiencing negative emotion, these differences in music selection highlight the importance of context in understanding how music helps people cope with different kinds of negative events.

The observed skew in the musical characteristics suggests that participants may have been listening to music that was similar to their affective state. This is consistent with the finding that young people listen to mood congruent music, likely to explore and process their negative emotional states (Dingle et al., 2019). On the level of affect regulation, the skew in musical characteristics is also consistent with findings that demonstrate high musical energy does not exasperate physiological arousal, and can facilitate positive emotional experiences (Sharman and Dingle, 2015; Thompson et al., 2018). Different people may use different music to achieve the same intended outcome—in this case, coping with stress. The audio features of the music are only part of the story, as situations, and the listener's response all play a role in some form (Hargreaves, 2012; Juslin, 2013; Baltazar, 2019). It is important to consider however that listener relationships with a selected piece of music can heighten a negative state when it is associated with negative memories, or aversive conditioned responses (Larwood and Dingle, 2021). In sum, the findings of our study broadly align with the notion that music which is affectively negative and arousing is not counter to hedonic goals.

Given the short timeframe for setting up this study during the first wave of COVID-19, there are limitations in the measurement approach and future research should refine and further explore the ideas started in this study. One limitation of the present study, although outside of its key aims, was the lack of measurement surrounding the physical location of participants. It is unclear whether students were in Australia, or their home countries learning online, and whether this had a differential impact on the stressors related to COVID-19. What was clear was that well-being in the present sample was lower than established norms in the earlier UK sample (Ng Fat et al., 2017), highlighting the impact of COVID-19 on well-being. In addition, when considering the trend toward coping songs being moderate in arousal and negatively valenced, future research may wish to explore how this varies with BRECVEMA mechanisms (Juslin, 2013), mechanisms by which music is being used to find social support or surrogacy (Hanser et al., 2016; Schäfer and Eerola, 2020; Schäfer et al., 2020), and how music use may be hedonic or eudaimonic in the stressful context of COVID-19 (Eden et al., 2020). This line of research would be particularly useful in considering how individuals can best curate their listening not only in the context of the current pandemic and associated social distancing but also more generally for times of increased stress and limited social context. Furthermore, several of the coping strategies examined, such as music listening and exercise, can be used simultaneously under many circumstances. While not possible to look at in the context of the present study, future research might examine how multiple coping strategies could be used in an additive way during the pandemic in order to facilitate stress management.

The current study examined music listening during the COVID-19 pandemic, and in line with previous research with young people, found music listening, among other strategies, was an effective stress management strategy for both domestic and international University students. Although domestic and international students experienced different levels of stress resulting from COVID-19, their well-being was comparable, and students who found music listening to be an effective coping strategy reported better well-being. Furthermore, songs nominated by students as helpful for managing stress tended to be negative in valence, and moderate in energy; however, there were few similarities between the songs chosen. This suggests that there is no one-size-fits-all solution to music listening—even in the unique context of a global pandemic, the type of music used for coping may not matter if the listener perceives the potential benefits of music listening.

## Data Availability Statement

The datasets generated for generated for this study can be found in online repositories. The names of the repository/repositories and accession number(s) can be found at: https://github.com/joellarwood/covid_music.

## Ethics Statement

The studies involving human participants were reviewed and approved by University of Queensland Health and Behavioral Sciences Low and Negligible Risk Ethics Committee. The patients/participants provided their written informed consent to participate in this study.

## Author Contributions

All authors conceived the study. DV wrote the first draft of the abstract, introduction, and discussion. JL wrote the first draft of the method and results. All authors approved and edited the final version of the manuscript.

## Conflict of Interest

The authors declare that the research was conducted in the absence of any commercial or financial relationships that could be construed as a potential conflict of interest.
