# Supplementary material for: Music Listening as a Strategy for Managing COVID-19 Stress in First-Year University Students
Source: Front Psychol. 2021 Apr 1;12:647065. doi: 10.3389/fpsyg.2021.647065 (PMC8047110; doi:10.3389/fpsyg.2021.647065)
Supplement: Supplementary file 1 [file Data_Sheet_1.DOCX]

**University Student Wellbeing Survey**

**PART A – DEMOGRAPHICS, ENROLMENT and HEALTH SERVICE USE**

**1. What is your gender?**

□ Male

□ Female

□ Prefer not to say

**2. What is your age in years?** _________

**3. What is your current relationship status?**

□ Single (never married)

□ In a relationship (not living together)

□ In a relationship (living together)

□ Married

□ Divorced

□ Other _____________________

Branch to next question if no relationship

**If in a relationship, does that person live in Brisbane?**

□ Yes

□ No

**4. Which UQ school are you enrolled in?**

□ Business

□ Pharmacy

□ Psychology

□ ICTE

□ Other, please write below _____________________

**5. Are you currently enrolled as a domestic or international student?**

□ Domestic

□ International

**6. What country/countries do you currently hold citizenship in? _________________________**

**7. What cultural or ethnic group do you belong to? _____________________**

**8. What is your current living situation?**

□ Living with family

□ Living alone

□ Share-house

□ UQ College

□ Student accommodation (shared)

□ Student accommodation (studio)

**9. What is your current circumstance in terms of the restrictions of COVID-19?**

□ In self-isolation because you have been infected

□ In self-isolation because you have been in close contact with a confirmed case

□ Feeling sick or showing symptoms of coronavirus

□ Staying at home for study/work

□ Still going out for study/work

□ Other, please specify___________

**PART B - CAUSES OF STRESS**

**Please indicate from 1 (Strongly disagree) to 5 (Strongly agree) whether the following issues are a cause of stress to you. If any do not apply to you, please answer ‘Neutral’.**

|  | Strongly disagree | Disagree | Neutral | Agree | Strongly agree | |
| --- | --- | --- | --- | --- | --- | --- |
| 1. Speaking up in the classroom/online (e.g. asking questions, tutorial discussions) |  |  |  |  |  | |
| 1. Issues with presenting in class/online |  |  |  |  |  | |
| 1. Issues with individual written assignments |  |  |  |  |  | |
| 1. Issues with group assignments/projects |  |  |  |  |  | |
| 1. Issues with assessment-related procedures (e.g. seeking extensions) |  |  |  |  |  | |
| 1. Issues with studying for exams |  |  |  |  |  | |
| 1. Issues with completing written exams in time (e.g. use of aids, dictionary, etc.) |  |  |  |  |  | |
| 1. Issues with time management of extra-curricular activities |  |  |  |  |  | |
| 1. Personal financial issues |  |  |  |  |  |  |
| 1. Environment and climate change |  |  |  |  |  |  |
| 1. Trying to maintain a healthy lifestyle |  |  |  |  |  |  |
| 1. Trying to maintain a healthy diet |  |  |  |  |  |  |
| 1. Trying to keep a good body image |  |  |  |  |  |  |
| 1. Family issues |  |  |  |  |  |  |
| 1. Issues around personal safety |  |  |  |  |  |  |
| 1. Friendship issues |  |  |  |  |  |  |
| 1. Relationship issues |  |  |  |  |  |  |
| 1. Mental health issues |  |  |  |  |  |  |
| 1. Personal health issues |  |  |  |  |  |  |
| 1. Issues with disclosing sexuality to others |  |  |  |  |  |  |
| 1. Issues finding paid work |  |  |  |  |  |  |
| 1. Issues in the workplace |  |  |  |  |  |  |
| 1. Issues with living arrangements |  |  |  |  |  |  |
| 1. Issues with domestic chores (e.g. housework, laundry, cooking) |  |  |  |  |  |  |
| 1. Issues with the health of others close to you |  |  |  |  |  |  |

**The following questions are specifically about stress specific to COVID-19**

**Please indicate from 1 (Strongly disagree) to 5 (Strongly agree) whether the following issues are a cause of stress to you. If any do not apply to you, please answer ‘Neutral’.**

|  | Strongly disagree | Disagree | Neutral | Agree | Strongly agree |
| --- | --- | --- | --- | --- | --- |
| 1. Anxiety about being exposed to COVID-19 infection |  |  |  |  |  |
| 1. Travel restrictions |  |  |  |  |  |
| 1. Social distance restrictions |  |  |  |  |  |
| 1. Avoiding crowded places due to risk of exposure to COVID-19 |  |  |  |  |  |
| 1. I was required to self-isolate due to potential risk |  |  |  |  |  |
| 1. My study was disrupted |  |  |  |  |  |
| 1. My work was disrupted |  |  |  |  |  |
| 1. I have financial issues due to COVID-19 |  |  |  |  |  |
| 1. I’m worried about myself being infected |  |  |  |  |  |
| 1. I’m worried about my family members being infected |  |  |  |  |  |
| 1. I’m worried about my friends being infected |  |  |  |  |  |
| 1. Anxiety about reading reports related to COVID-19 in the news and social media |  |  |  |  |  |
| 1. Worry about running out of medical and other supplies / groceries |  |  |  |  |  |

Please describe any other ways in which you have been impacted by the COVID-19? _______________________________________

**Please indicate from 1 (Almost never) to 5 (Almost always) whether the statement is describing your emotional response in the context of COVID-19**

|  | Almost never | Sometimes | About half the time | Most of the time | Almost always |
| --- | --- | --- | --- | --- | --- |
| 1. I pay attention to how I feel. |  |  |  |  |  |
| 1. I have no idea how I am feeling. |  |  |  |  |  |
| 1. I have difficulty making sense out of my feelings. |  |  |  |  |  |
| 1. I am attentive to my feelings. |  |  |  |  |  |
| 1. I am confused about how I feel. |  |  |  |  |  |
| 1. When I’m upset, I acknowledge my emotions. |  |  |  |  |  |
| 1. When I’m upset, I become embarrassed for feeling that way. |  |  |  |  |  |
| 1. When I’m upset, I have difficulty getting work done. |  |  |  |  |  |
| 1. When I’m upset, I become out of control. |  |  |  |  |  |
| 1. When I'm upset, I believe that I will remain that way for a long time. |  |  |  |  |  |
| 1. When I'm upset, I believe that I'll end up feeling very depressed. |  |  |  |  |  |
| 1. When I'm upset, I have difficulty focusing on other things. |  |  |  |  |  |
| 1. When I'm upset, I feel ashamed with myself for feeling that way. |  |  |  |  |  |
| 1. When I'm upset, I feel guilty for feeling that way. |  |  |  |  |  |
| 1. When I'm upset, I have difficulty concentrating. |  |  |  |  |  |
| 1. When I'm upset, I have difficulty controlling my behaviors. |  |  |  |  |  |
| 1. When I'm upset, I believe that wallowing in it is all I can do. |  |  |  |  |  |
| 1. When I'm upset, I lose control over my behaviors. |  |  |  |  |  |

**PART C. COPING STRATEGIES**

**Please indicate how often you find the following effective for managing stress by yourself: If you don’t use these or they aren’t effective, use none of the time.**

|  | Do not use this strategy | | Not effective | | Rarely effective | | Sometimes effective | | Often effective | | Always effective | |
| --- | --- | --- | --- | --- | --- | --- | --- | --- | --- | --- | --- | --- |
| 1. Exercise | | □ | | □ | □ | | □ | | □ | | □ | |
| 1. Listen to music | | □ | | □ | □ | | □ | | □ | | □ | |
| 1. Tend to jobs around the house | | □ | | □ | □ | | □ | | □ | | □ | |
| 1. Rest, nap or sleep | | □ | | □ | □ | | □ | | □ | | □ | |
| 1. Try to control thoughts | | □ | | □ | □ | | □ | | □ | | □ | |
| 1. Evaluate or analyse the situation | | □ | | □ | □ | | □ | | □ | | □ | |
| 1. Put feelings in perspective | | □ | | □ | □ | | □ | | □ | | □ | |
| 1. Avoid thing (person) causing the mood | | □ | | □ | □ | | □ | | □ | | □ | |
| 1. Be alone | | □ | | □ | □ | | □ | | □ | | □ | |
| 1. Use relaxation techniques (e.g. deep breathing, muscle relaxation, mindfulness etc.) by myself | | □ | | □ | □ | | □ | | □ | | □ | |
| 1. Engage in stress management (e.g. get organised, plan ahead, make lists) | | □ | | □ | □ | | □ | | □ | | □ | |
| 1. Religious activity by myself | | □ | | □ | □ | | □ | | □ | | □ | |
| 1. Change location (e.g. go for a drive or go outside) | | □ | | □ | □ | | □ | | □ | | □ | |
| 1. Call someone on the phone | | □ | | □ | | □ | | □ | | □ | | □ |
| 1. Use the internet for social communication | | □ | | □ | | □ | | □ | | □ | | □ |

1. Please consider the coping strategies above and indicate from 1 to 15, which is the most effective one for you? ____
2. If listening to music is an effective strategy for you, please write the name of a piece of music and artist that you have found to help manage your stress: artist________ song _______

**PART D. SOCIAL GROUPS AND SUPPORT**

**Please indicate from 1 (Strongly disagree) to 5 (Strongly agree) whether the following statements apply to you.**

|  | Strongly disagree | Disagree | Neutral | Agree | Strongly agree |  |
| --- | --- | --- | --- | --- | --- | --- |
| 1. I belong to lots of different groups |  |  |  |  |  | |
| 1. I join in the activities of lots of different groups |  |  |  |  |  | |
| 1. I am friendly with people in lots of different groups |  |  |  |  |  | |
| 1. I have strong ties with lots of different groups |  |  |  |  |  | |
| 1. I feel a sense of belonging with my family |  |  |  |  |  | |
| 1. I feel a sense of belonging with my Australian friends |  |  |  |  |  | |
| 1. I feel a sense of belonging with my friends at home |  |  |  |  |  | |
| 1. I feel a sense of belonging with my school of enrolment |  |  |  |  |  | |
| 1. I feel a sense of belonging as a student at the University of QLD |  |  |  |  |  | |

**Please indicate your answer to the following questions from 1 (Hardly ever) to 3 (Often).**

|  | Hardly Ever | Some of the Time | Often |
| --- | --- | --- | --- |
| 1. How often do you feel that you lack companionship? |  |  |  |
| 1. How often do you feel left out? |  |  |  |
| 1. How often do you feel isolated from others? |  |  |  |

**The following questions about ‘How others view you’ and ‘Homesickness’ are only for international students, branch to the next part if you are a domestic student.**

**The following questions ask about how others in Australian society view people like you (i.e., international students) in the context of the COVID-19 pandemic.**

**Please indicate from 1 (Strongly disagree) to 5 (Strongly agree) whether the following statements apply to you.**

|  | Strongly disagree | Disagree | Neutral | Agree | Strongly agree |
| --- | --- | --- | --- | --- | --- |
| 1. I feel people look down on us international students |  |  |  |  |  |
| 1. People like us are treated with respect |  |  |  |  |  |
| 1. Others hold prejudice against us international students |  |  |  |  |  |
| 1. People like us are generally given a chance of success |  |  |  |  |  |
| 1. People like us face discrimination |  |  |  |  |  |
| 1. We international students are treated the same as everyone else |  |  |  |  |  |

**Please indicate from 1 (Very often) to 5 (Never) whether the following statements apply to you.**

|  | Very often | Often | Sometimes | Rarely | Never |
| --- | --- | --- | --- | --- | --- |
| I want to go back to my home city/country |  |  |  |  |  |
| I think about what I would do if I were back home |  |  |  |  |  |
| I feel homesick |  |  |  |  |  |
| I miss my friends and family back home |  |  |  |  |  |

**PART E. STUDENT WELLBEING**

**Below are some statements about feelings and thoughts. Please tick the box that best describes your experience of each over the last 2 weeks.**

|  | None of the time | Rarely | Some of the time | Often | All of the time |
| --- | --- | --- | --- | --- | --- |
| 1. I’ve been feeling optimistic about the future |  |  |  |  |  |
| 1. I’ve been feeling useful |  |  |  |  |  |
| 1. I’ve been feeling relaxed |  |  |  |  |  |
| 1. I’ve been dealing with problems well |  |  |  |  |  |
| 1. I’ve been thinking clearly |  |  |  |  |  |
| 1. I’ve been feeling close to other people |  |  |  |  |  |
| 1. I’ve been able to make up my own mind about things |  |  |  |  |  |

**For the questions below, please tick the “Yes” box if you have had this symptom in the last 30 days.**

|  | No | Yes |
| --- | --- | --- |
| 1. Do you often have headaches? |  |  |
| 1. Is your appetite poor? |  |  |
| 1. Do you sleep badly? |  |  |
| 1. Are you easily frightened? |  |  |
| 1. Do your hands shake? |  |  |
| 1. Do you feel nervous? |  |  |
| 1. Is your digestion poor? |  |  |
| 1. Do you have trouble thinking clearly? |  |  |
| 1. Do you feel unhappy? |  |  |
| 1. Do you cry more than usual? |  |  |
| 1. Do you find it difficult to enjoy your daily activities? |  |  |
| 1. Do you find it difficult to make decisions? |  |  |
| 1. Is your daily work suffering? |  |  |
| 1. Are you unable to play a useful part in life? |  |  |
| 1. Have you lost interest in things? |  |  |
| 1. Do you feel that you are a worthless person? |  |  |
| 1. Has the thought of ending your life been on your mind? |  |  |
| 1. Do you feel tired all the time? |  |  |
| 1. Do you have uncomfortable feelings in the stomach? |  |  |
| 1. Are you easily tired? |  |  |

**Please indicate from 1 (Strongly disagree) to 5 (Strongly agree) whether you agree or disagree with the following statements.**

|  | Strongly disagree | Disagree | Neutral | Agree | Strongly agree |
| --- | --- | --- | --- | --- | --- |
| 1. I am satisfied with my body weight and shape. |  |  |  |  |  |
| 1. I engage in exercise several times a week (e.g. exercise at home, walk outside). |  |  |  |  |  |
| 1. I eat a healthy, balanced diet. |  |  |  |  |  |
| 1. I tend to put studying off (procrastinate). |  |  |  |  |  |
| 1. I am coping with my study. |  |  |  |  |  |
| 1. I have good self-esteem. |  |  |  |  |  |

**Please circle your answer to the following questions below.**

| 1. How often do you have a drink containing alcohol? | Never | Monthly or less | 2-4 times per month | 2-3 times per week | 4+ times per week |
| --- | --- | --- | --- | --- | --- |
| 1. How many standard drinks of alcohol do you drink on a typical day when you are drinking? | 1-2 | 3-4 | 5-6 | 7-9 | 10+ |
| 1. How often do you have 5 or more drinks on one occasion? | Never | Less than monthly | Monthly | Weekly | Daily or almost daily |

**Do you need help?**

It is normal to experience some worry or concern during this period, but if those feelings start to affect your mental or physical wellbeing, it is recommended that you seek support from a health professional.

If you want to speak to someone, please do not hesitate to contact the following services (you can screen shot this page):

Campus Security (3365 3333) for Emergency Services

Student Services: [student.services@uq.edu.au](mailto:student.services@uq.edu.au)

Student Counselling & Crisis Line (1300 851 998)

UQ Students Virtual Village <https://life.uq.edu.au/village>

UQ Psychology Clinic (telehealth sessions): <https://clinic.psychology.uq.edu.au/therapies-and-services/referrals>

If you need some help with your health, UQ Health Care can also be contacted for appointments. Their team of GPs, nurses, allied health professionals and specialists provide care across our five medical clinics in Ipswich, Annerley, Meadowbrook, St Lucia and Gatton. Contact UQ Health Care:

St Lucia: (07) 3365 6210

Gatton: (07) 5460 1396

Annerley / PACE: (07) 3346 1122

Ipswich: (07) 3381 1800

Meadowbrook: (07) 3489 9400

**Thank you for taking the time to complete this survey!**

Please enter your email address here to be given your 1 hour credit (Psychology students only) __________

OR to go into the draw to win one of 10 JB Hi-Fi vouchers each worth $30 (Any students. Psychology students please select only one option) ___________
